# Editorial: Molecular basis of seed longevity

**DOI:** 10.3389/fpls.2023.1138139

**Published:** 2023-01-30

**Authors:** Ewa Marzena Kalemba, Françoise Corbineau, S Prashant Jeevan Kumar

**Affiliations:** ^1^ Institute of Dendrology, Polish Academy of Sciences, Kórnik, Poland; ^2^ Biologie des Semences, Unité Mixte de Recherche (UMR), Institut de Biologie Paris-Seine (IBPS), Sorbonne Université, Paris, France; ^3^ Biotechnology Department, ICAR-Directorate of Floricultural Research, Pune, Maharashtra, India

**Keywords:** accelerated aging, lipidomics, legumes, nitric oxide, storability, sorption isotherm, transcription factor

Seeds, in particular of orthodox type, represent the most important stage in plant reproduction and play an essential role in plant spread. Seeds vary in their germination characteristics including dormancy, longevity, and seed sensitivity to temperature. Seed longevity depends on the seeds themselves as well as on the storage conditions, and prolonging seed life is a global challenge for the conservation of plant biodiversity. Genetically, seed longevity is a polygenic trait, but physiologically requires the coordination of many biological processes that shape the stability of seeds in the soil and their *ex situ* storability (Zinsmeister et al., 2020).

To meet targets in biodiversity conservation, genebanking programs, agriculture, horticulture and reforestation initiatives, it is necessary to characterize the molecular basis of seed viability. Knowledge of the reasons behind poor seed longevity is useful for optimizing storage protocols in seed banks, enabling sustained seed viability for prolonged periods of time (De Vitis et al., 2020). Seed longevity, also known as storability, is defined as seed viability upon seed dry storage. Experimental studies concerning the effect of temperature and moisture content (MC) on seed survival defined optimal seed storage conditions that turned out to be effective at improving storability of seeds of many species.

Although seed longevity is definitely an attribute of all desiccation-tolerant (orthodox) seeds, it nonetheless varies within and among species, as well as in seed lots harvested in different years. In contrast, desiccation sensitive (recalcitrant) seeds rapidly lose viability after dehydration (Roberts, 1973; Priestley, 1986; Zinsmeister et al., 2020). Upon extended storage, seed vigor decreases, and seeds of both categories deteriorate over time, losing their germination ability. In the context of combating climate change, understanding internal mechanisms acting in seeds at the molecular level and contributing to seed longevity is becoming a critical and urgent issue. This special volume brings together five articles (three original research and two review manuscripts) that cover the subject of seed longevity implicating gene regulatory networks, seed structure and composition, plant hormones, reactive oxygen species (ROS), nitric oxide (NO), membrane lipids, and proteins acting on gene expression as factors contributing to seed quality.

Sustainable agriculture provides instructions for breeding programs on how to make crop production more efficient and less detrimental to the environment, but it still depends on the quality of seeds used for farming. Legumes are a rich source of proteins, vitamins, and fiber and are an important component of a healthy human diet. In the present issue, Ramtekey et al. provide a comprehensive overview of molecular basis of seed longevity in legumes with an emphasis on the protective and repair mechanisms, hormonal signaling, and homeostasis between ROS and antioxidants. The authors give attention to heat shock proteins (HSPs) and late embryogenesis abundant (LEA) protein accumulation associated with longevity, genes related to chlorophyll metabolism, and repair of damaged DNA and membrane components. Soybean is an important species among crop legumes that exhibit seed longevity problems that detrimentally affect its cultivation. The study carried out by Lin et al. (2022) reveals how membrane lipid components dynamically change during the aging process in soybean. Using lipidomics, the authors selected diacylglycerol 36:4; phosphatidylcholine 34:2, 36:2, and 36:4; and phosphatidylethanolamine 34:2 as novel molecular hallmarks of soybean seed aging.

Studies on *Arabidopsis thaliana* have led to many discoveries in modern plant biology, including that in seed science. Recently, a genome-wide association study attributed differences in *A. thaliana* seed longevity to sequence polymorphism in enzyme genes that participate in the metabolism of ROS and to several transcription factor genes involved in seed coat development (Renard et al., 2020). The abovementioned molecular mechanisms involved in aging were addressed in the study of Niñoles et al. who leveraged the “elevated partial pressure of oxygen” treatment to accelerate seed aging in *A. thaliana* seeds. The authors identified DOF4.1, a C2H2-type zinc-finger transcription factor from the DNA binding with one finger family, as a novel regulator of seed longevity, providing new insights into transcriptional regulation of seed coat development and seed storage protein accumulation. Interestingly, seed permeability of *dof4.1* mutants was reduced and linked with their higher longevity. In contrast, elevated electrolyte leakage was reported in aged soybean seeds (Lin et al., 2022). Both studies indicated the importance of the maintenance of functional lipid barriers in seed longevity.

Seed longevity is often studied using artificial accelerated aging that significantly shortens the required time to conduct seed quality research as compared to waiting for natural aging to occur. This approach was used not only in the aforementioned study by Niñoles et al. but also in artificially aged soybean (Lin et al., 2022) and apple seeds Ciacka et al. The role of NO was investigated in the preservation of apple seed longevity. Ciacka et al. revealed the positive role of NO in delaying the negative effects of aging *via* modification of the expression of *Hsp* (*Hsp70B* and *Hsp70C*) and *Lea* (*Lea2a*) genes, whose products are considered to be determinants of seed longevity Ramtekey et al. The authors propose that NO application may stimulate the use of stored mRNA, lower the oxidation level of RNA, and stimulate *de novo* synthesis of mRNA. Additionally, increased *meta*-tyrosine (m-Tyr) concentration was observed and linked to the progression of aging Ciacka et al. Therefore, m-Tyr was proposed as a new marker of cellular aging.

MC is a seed attribute related to the seed quality. Seed harvesting and extraction, followed by processing, drying, and storage, all externally affect seed MC and, therefore, seed longevity. These factors are addressed in a review article by Hay et al, in which it is explained how sorption isotherms can help to understand the dependence of seed longevity on seed drying and storage conditions. A seed isotherm describes the relationship between MC and water activity, thereby indicating how water is bound within the seed tissues. The authors characterize various sorption models and explain how these models should be interpreted, revealing their utility for approximating seed longevity in genebanks.

Overall, the compilation of articles included in this special issue demonstrates the complexity of seed longevity traits and highlights the significance of investigating at the molecular level the roles of environmental and endogenous cues that regulate seed quality. Together, these studies contributed to a better understanding of factors affecting seed viability and, at the same time, provide perspectives for the future and raise new scientific questions that need to be answered in the coming years for successful germplasm conservation.

## Author contributions

EK wrote the first draft of the manuscript. EK, FC, and SK reviewed and edited the manuscript. All authors read, and approved the submitted version.
